# MicroRNAs in the etiology of colorectal cancer: pathways and clinical implications

**DOI:** 10.1242/dmm.027441

**Published:** 2017-03-01

**Authors:** Ashlee M. Strubberg, Blair B. Madison

**Affiliations:** Division of Gastroenterology, Washington University School of Medicine, Washington University, Saint Louis, MO 63110, USA

**Keywords:** Cancer, Colon, Colorectal, Rectal, Tumorigenesis, microRNA

## Abstract

MicroRNAs (miRNAs) are small single-stranded RNAs that repress mRNA translation and trigger mRNA degradation. Of the ∼1900 miRNA-encoding genes present in the human genome, ∼250 miRNAs are reported to have changes in abundance or altered functions in colorectal cancer. Thousands of studies have documented aberrant miRNA levels in colorectal cancer, with some miRNAs reported to actively regulate tumorigenesis. A recurrent phenomenon with miRNAs is their frequent participation in feedback loops, which probably serve to reinforce or magnify biological outcomes to manifest a particular cellular phenotype. Here, we review the roles of oncogenic miRNAs (oncomiRs), tumor suppressive miRNAs (anti-oncomiRs) and miRNA regulators in colorectal cancer. Given their stability in patient-derived samples and ease of detection with standard and novel techniques, we also discuss the potential use of miRNAs as biomarkers in the diagnosis of colorectal cancer and as prognostic indicators of this disease. MiRNAs also represent attractive candidates for targeted therapies because their function can be manipulated through the use of synthetic antagonists and miRNA mimics.

## Abstract (precis)

**Summary:** This Review provides an overview of some important microRNAs and their roles in colorectal cancer.

## Introduction

**Fig. 1.**
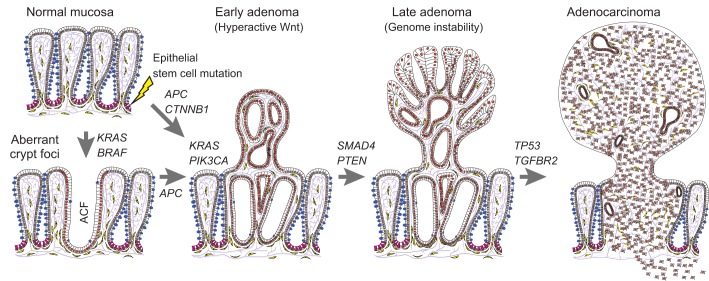
**Step-wise tumorigenesis in colorectal cancer.** Cartoon of the large intestine showing the structure of the normal colonic mucosa, with many mucous-secreting goblet cells (blue) at top left. Tumorigenesis begins with the mutation of intestinal epithelial stem cells (shown in magenta) in the colon or rectal mucosa, with mutations often occurring first in the *APC*, *KRAS* or *BRAF* genes. Mutations in *BRAF* or *KRAS* (in the absence of Wnt pathway mutations) are often associated with the formation of aberrant crypt foci (ACF). Most adenomas are associated with mutations in Wnt pathway components, such as *APC* or *CTNNB1*, which result in hyperactivation of Wnt signaling in early adenomas. Deregulation of Wnt signaling often co-occurs with mutations in *KRAS*, *PIK3CA*, or other mutations, leading to activation of the PI3K-Akt signaling cascade. Adenomas then progress with additional mutations (e.g. *SMAD4*) and frequently acquire genomic instability. Lastly, mutations in *TP53* and *TGFBR2* are associated with later stages of cellular transformation and with invasive characteristics of adenocarcinomas. Official human gene symbols and full names: *AKT*, AKT serine/threonine kinase 1; *APC*, adenomatous polyposis coli or Wnt signaling pathway regulator; *BRAF*, B-Raf proto-oncogene, serine/threonine kinase; *KRAS*, Kirsten rat sarcoma viral oncogene homolog or proto-oncogene and GTPase; *PIK3CA*, phosphatidylinositol-4,5-bisphosphate 3-kinase catalytic subunit alpha; *SMAD4*, Mothers against decapentaplegic homolog family member 4; *TGFBR2*, transforming growth factor beta receptor 2; *TP53*, tumor protein p53.

Colorectal cancer (CRC) is the fourth leading cause of cancer-related deaths worldwide (McGuire, 2016) and the second leading cause of cancer-related deaths in the USA (https://www.cdc.gov/cancer/colorectal/). Most cases of CRC are sporadic, although 20-30% of affected individuals carry inherited mutations (Da Silva et al., 2016; Hahn et al., 2016). CRC is generally classified into five stages, 0 to IV, characterized by submucosal invasion (stage I), penetration of the outer colonic wall (II), lymph node invasion (III) and metastasis (IV). The morphological changes and major mutations (in key tumor suppressor genes such as *APC* and *TP53*) that are involved in the formation of pre-cancerous lesions (or adenomas) have been determined via the examination of biopsies and are thus generally well defined (Fig. 1). The entire process of CRC tumorigenesis is slow – it is estimated to take nearly two decades for a tumor to develop (Jones et al., 2008) – and endoscopy has proven to be effective for early detection and removal of adenomas and tumors (Rabeneck et al., 2010). Death rates from colorectal cancer have declined over the past 20 years, largely thanks to early detection; however, CRC incidence still remains high (Siegel et al., 2015) and new treatments have been lagging. Treatment usually entails surgical removal of tumors, which may be followed with chemotherapy and/or targeted biologics for stage III and IV tumors (Graham and Cassidy, 2012). New drugs that have recently been approved are primarily biologics that target tumor angiogenesis (VEGF, the vascular endothelial growth factor and its receptor, VEGFR) or the epidermal growth factor receptor (EGFR, see Glossary, Box 1) (Köhne, 2014). However, these drugs have had limited success, and in the case of EGFR inhibitors, oncogenic mutations in *KRAS* (occurring in about 40% of CRCs) confer resistance (Gong et al., 2016). Therapies targeting microRNAs (miRNAs) or their pathways may provide new or complementary targets for therapeutic and preventative applications.

**Box 1. Glossary of selected terms and genes/proteins involved in CRC**

**Argonaute (Ago):** A protein that is a critical component of the RNA-induced silencing complex (RISC) that mediates inhibitory effects on mRNA translation by miRNAs. Four Ago proteins have been characterized in mammals (AGO1-AGO4), which all appear capable of functioning to target and silence mRNAs via miRNAs and the RISC.

***CTNNB1*:** Gene that encodes β-catenin, a transcriptional effector in Wnt signaling that binds and interacts with the DNA-binding transcription factors TCF4 and LEF1. β-catenin also binds to the intracellular domain of E-cadherin at adherens junctions, distinct from its role in Wnt signaling.

**Dextran sulfate sodium (DSS)-induced colitis model:** DSS oral administration to mice causes intestinal epithelial damage and cell death, leading to a compromised barrier and severe inflammation. This inflammation resembles human ulcerative colitis (UC), with increases of pro-inflammatory cytokines, such as TNFα, IL-1α/β, IL-6 and IL-18 (Chassaing et al., 2014).

**Dicer:** An RNase III family enzyme capable of cleaving double-stranded RNA. This enzyme catalyzes the second cleavage event during miRNA biogenesis whereby it cleaves the pre-miRNA ‘hairpin’ structure near the terminal loop region, to remove this structure to generate a double-stranded miRNA**.**

**Digital PCR:** An approach for the precise nucleic acid quantification by directly counting the total number of target molecules through multiplexed nanoliter-sized reactions.

**Epithelial-to-mesenchymal transition (EMT):** The morphological and phenotypic change of an epithelial cell into a fibroblast-like cell. This process appears necessary for the migration of an epithelial-derived cancer through the basement membrane, into and out of the vasculature, culminating in the metastasis of malignant cells to distant organs.

**Fecal occult blood test (FOBT):** Assay used to detect occult blood in stool samples that may indicate the presence of colorectal polyps or cancer.

**Hypermutated colorectal cancers:** This category of CRCs account for about 15% of all cases and is characterized by a high rate of mutations (>12 per 10^6^ bases or >180 per exome), microsatellite instability (MSI), and defects in DNA mismatch repair. However, these cancers typically possess a stable diploid genome and lack large chromosomal translocations and deletions (Cancer Genome Atlas Network, 2012; Guinney et al., 2015).

**IL-6/STAT3 signaling:** IL-6 is a pro-inflammatory cytokine produced by T-cells and macrophages in response to infection or tissue damage. Through the IL6R-GP130 heterodimeric receptor, and associated Janus-associated kinase (JAK) proteins, IL-6 activates the transcription factor STAT3, which triggers the transcription of genes involved in the acute phase response, such as C-reactive protein, serum amyloid A, and fibrinogen. Activation of the IL-6/STAT3 signaling cascade is a driver of tumorigenesis through effects on the intestinal epithelium by enhancing cell survival and proliferation (Quante et al., 2013). Cancer associated fibroblasts in CRC are also a source of IL-6 (Huynh et al., 2016).

**Let-7:** Let-7 miRNAs comprise one of the largest miRNA families in mammals, with 12 genes encoding 8 unique miRNAs (Kamanu et al., 2013). The Let-7 miRNA family is implicated in maintaining differentiation and preventing tumorigenesis across multiple tissue types (Takamizawa et al., 2004; Johnson et al., 2007; Sampson et al., 2007; Shell et al., 2007; Boyerinas et al., 2008).

**Lipopolysaccharide (LPS):** Large molecule found in the outer membrane of gram-negative bacteria consisting of a polysaccharide unit covalently linked to a disaccharide that is linked to multiple fatty acids. LPS is highly immunogenic endotoxin that binds to and activates the Toll-like receptor 4 (TLR4).

**MET (mesenchymal-to-epithelial transition):** The morphological and phenotypic change of a fibroblast-like cell into a epithelial cell. This process appears necessary for metastasis to distal organs, although the role of such a process in CRC metastasis remains to be fully characterized.

**NF-κB (NFΚB):** A transcription factor formed by homodimers and heterodimers of five gene products that is activated downstream of stressors (free radicals), pro-inflammatory cytokines (such as TNFα, IL-1, and LT-β), and microbial products (via Toll-like receptors). The activation of NFΚB represses apoptosis (Kucharczak et al., 2003), regulates the immune responses in the gut (Wullaert et al., 2011) and plays a role in fueling CRC tumorigenesis (Ben-Neriah and Karin, 2011; Vaiopoulos et al., 2013).

**Notch signaling in the intestine:** The NOTCH1 and NOTCH2 transmembrane receptors are expressed in the IESCs and transmit signals from Notch ligands, such as DLL1, DLL4 and JAG1 (expressed in adjacent cells). Notch signaling is required to maintain stem cell fate and proliferation in the intestine (Pellegrinet et al., 2011), and is frequently activated in CRC (Chu et al., 2011; Rodilla et al., 2009).

**NUMB:** A membrane-localized protein asymmetrically distributed following stem cell division that plays an important role negatively regulating Notch. It also can destabilize β-catenin in a Notch-dependent, but Notch ligand-independent manner (Kwon et al., 2011).

**PGE2 (prostaglandin E2):** A pro-inflammatory prostaglandin that binds to the EP2 and EP4 receptors on T cells, dendritic cells, and intestinal epithelial cells (Dorsam and Gutkind, 2007). PGE2 can activate Wnt signaling and the KRAS-MAPK signaling cascade through SRC (Buchanan and DuBois, 2006), with phenotypic augmentation of angiogenesis, tumor proliferation, and metastasis (Dorsam and Gutkind, 2007).

**TNFα (tumor necrosis factor alpha):** A pro-inflammatory cytokine produced by activated macrophages, dendritic cells, and T cells, most frequently downstream of TLR activation. TNFα is a major contributer to the pathogenesis of inflammatory bowel disease, and stimulates angiogenesis, the production of other pro-inflammatory cytokines, and can trigger intestinal epithelial cell death**.**

MiRNAs are small, single-stranded RNAs of 21-23 nucleotides (nt) in length that repress mRNA translation and trigger mRNA degradation (Lin and Gregory, 2015; Towler et al., 2015). Their biogenesis involves several steps (see Box 2) and their functions can be post-transcriptionally modulated via the regulation of their biogenesis, interaction with targets, degradation and sequestration from other mRNAs (Ha and Kim, 2014). Defects in miRNA processing often appear to be associated with tumorigenesis (Lambertz et al., 2010; Sekine et al., 2009). These studies suggest that, in the context of global miRNA depletion, the loss of tumor-suppressive miRNAs (known as anti-oncomiRs) may have a greater effect on driving tumorigenesis than does the depletion of oncogenic miRNAs (known as oncomiRs). This phenomenon is particularly evident in mouse studies that have investigated the consequences of inactivating *Dicer1* (Lambertz et al., 2010; Sekine et al., 2009), which is required for the processing of almost all miRNAs (see Boxes 1 and 2). Human *DICER1* also appears to have a tumor-suppressive role in CRC cell lines (Iliou et al., 2014), and in other cancers, suggesting that miRNA biogenesis is essential for repressing tumorigenesis.

**Box 2. MicroRNA biogenesis**

**Figure:**
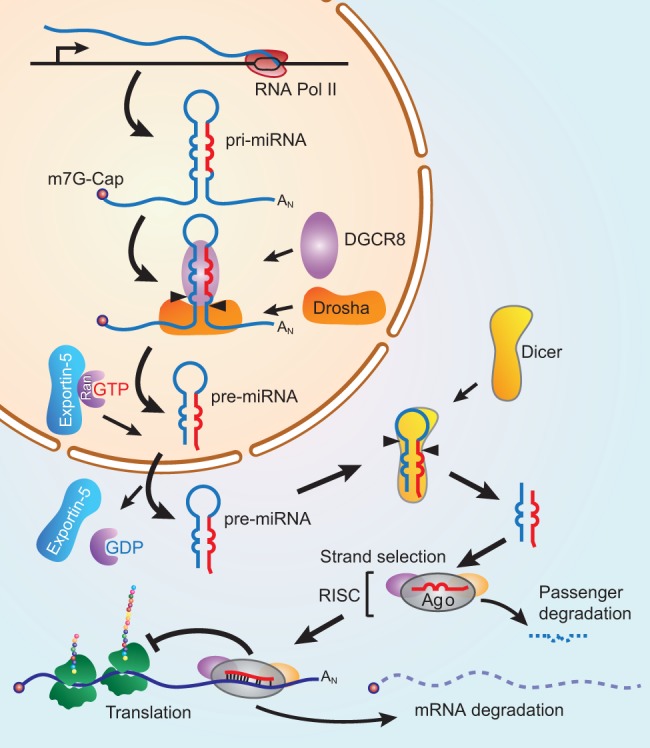

The biogenesis of miRNAs begins with the RNA polymerase II-mediated transcription of RNAs that are capped and polyadenylated. These primary miRNAs (pri-miRNAs) then undergo cleavage by the microprocessor complex (consisting of the RNase III nuclease Drosha and RNA-binding protein DGCR8) to generate short hairpin-shaped structures of 60-90 nucleotides (nt), called pre-miRNAs. These pre-miRNAs are exported from the nucleus by Ran/exportin-5 in a GTP-dependent manner to then be further processed in the cytoplasm by Dicer, also an RNase III nuclease, to generate 21-23 nt double-stranded miRNAs. MiRNAs are then loaded into a functional RNA-induced silencing complex (RISC) with an Argonaute (Ago) protein (e.g. AGO2) (see Glossary, Box 1). During this loading, a process called strand selection segregates the ‘guide’ strand (or miR, in red) from the ‘passenger’ (or miR*, in blue) strand. Within the RISC, the guide strand base pairs with complimentary sequences in the 3′UTR of target mRNAs, usually at positions 2-8 in the miRNA (the seed sequence). This interaction then triggers the repression of translation and ultimate degradation of the target mRNA. When investigators state that miRNAs directly inhibit a target, this refers to the repressive action of a miRNA on a specific mRNA via the RISC.

**Fig. 2.**
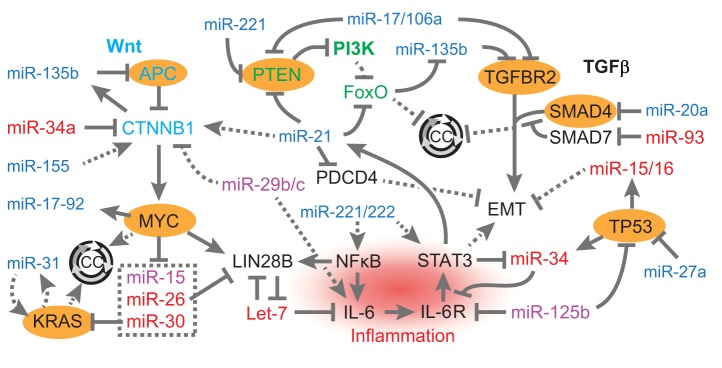
**Genes frequently mutated in colorectal cancer and their relationships with miRNAs.** Genes frequently mutated in CRC (highlighted in orange) regulate and are regulated by miRNAs. Oncogenic miRNAs are depicted in blue, tumor-suppressive miRNAs in red, and miRNAs with reported pleiotropic effects in purple. Direct relationships are shown with solid lines, while indirect relationships are illustrated with dotted lines. The Wnt pathway is augmented by miR-135b, miR-21 and miR-155, and inhibited by miR-34a, miR-29b/c. Downstream of Wnt, MYC transcriptionally activates the miR-17-92 locus, but represses expression of miR-15, miR-26 and miR-30. KRAS augments expression of miR-31. MYC and KRAS promote cell cycle progression (CC, circular arrows). In the PI3K pathway, which is negatively regulated by PTEN, miR-135b is augmented by PI3K inhibition of FoxO transcription factors (FOXO1 and FOXO3A), which represses cell cycle progression. MiR-221, miR-21 and miR-17/106 enhance activation of PI3K signaling by repressing negative regulators of this pathway. MiRNAs also modulate inflammatory pathways mediated by the transcription factors NFΚB and STAT3 by directly inhibiting IL-6 (via Let-7 miRNAs, which are inhibited by LIN28B) or the IL-6 receptor (via miR-34 and miR-125b). MiR-221/222 and miR-29b/c can also augment this pathway via indirect stimulatory effects on IL-6, NFΚB, and STAT3. The TGF-β pathway, which is important for repressing cellular proliferation and cell cycle progression is also antagonized by several miRNAs, including miR-17/106, miR-135b, and miR-20a through effects on TGFBR2 and SMAD4. The miRNA miR-93 can stimulate the TGF-β pathway by repressing the inhibitory SMAD7, although the effect of miR-93 is inhibitory of Wnt signaling through inhibition of SMAD7, which can augment nuclear accumulation of β-catenin. Lastly, several miRNAs have effects on EMT in CRC tumorigenesis, with miR-15/16 and miR-34 (which are transcriptionally activated by TP53) inhibiting this process, while miR-21 enhances EMT. References for the effects of these miRNAs can be found in Table 1 or in the main text. Official human gene symbols and full names: *APC*, adenomatous polyposis coli or WNT signaling pathway regulator; *CTNNB1*, β-catenin; *MYC*, v-myc avian myelocytomatosis viral oncogene homolog; *KRAS*, Kirsten rat sarcoma viral oncogene homolog or proto-oncogene and GTPase; PI3K, phosphatidylinositol-4,5-bisphosphate 3-kinase (*PIK3CA*, *PIK3CB*, *PIK3CD*, *PIK3CG*); PTEN, phosphatase and tensin homolog; FoxO, forkhead box O1 and O3a (*FOXO1* and *FOXO3A*); *PDCD4*, programmed cell death 4 (neoplastic transformation inhibitor); *LIN28B*, lineage-28 homolog B; NFΚB, nuclear factor kappa B (*NFKB1*, *NFKB2*, *REL*, *RELA*, *RELB*); *IL6*, interleukin 6; *IL6R*, interleukin 6 receptor; *STAT3*, signal transducer and activator of transcription 3; *TGFBR2*, transforming growth factor beta receptor 2; *SMAD4*, mothers against decapentaplegic homolog family member 4; *SMAD7*, mothers against decapentaplegic homolog family member 7; *TP53*, tumor protein p53.

In this Review, we expand on the most salient evidence linking individual miRNAs to the etiology of CRC, with a focus on the interaction of miRNAs with known oncogenic drivers and pathways. Information on direct targets of key miRNAs is listed in Table 1. Relationships among miRNAs and genes known to be involved in the initiation and progression of CRC are illustrated in Fig. 2. Genes highlighted are frequently inactivated (*APC*, *TGFBR2*, *TP53*, *SMAD4*, *PTEN*), constitutively activated (*KRAS*) or overexpressed (*MYC*) in CRC (Cancer Genome Atlas Network, 2012; Guinney et al., 2015). In CRC, the miRNA-mediated modulation of these genes has been found to regulate features of cellular transformation, while many miRNAs also function downstream of these factors. We will explore these relationships, and others that implicate miRNAs as critical modulators of CRC pathobiology and as potential therapeutic targets.

**Table 1.**
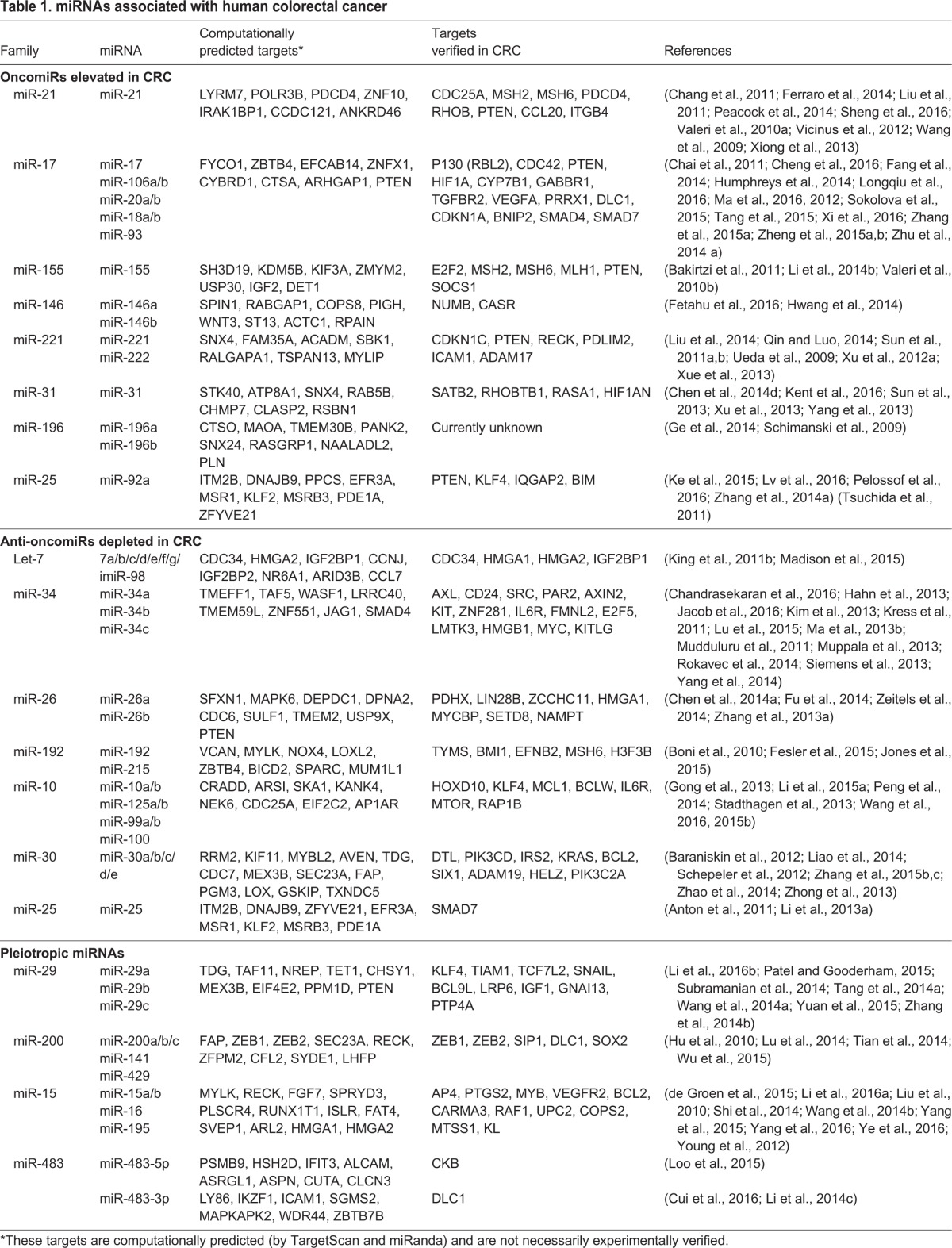
**miRNAs associated with human colorectal cancer**

## miRNA regulation of intestinal stem cells and CRC tumor-initiating cells

Several miRNA and miRNA pathways have been found to regulate normal intestinal epithelial stem cells (IESCs) in the mouse gut. IESCs are needed for replenishing epithelial cells, which are constantly turning over, much like skin cells. Cancer ‘stem cells’, by contrast, are defined by their tumor-initiating potential, and thus, may also be called tumor-initiating cells (TICs) – a nomenclature we will use for clarity. TICs typically exhibit frequent resistance to chemotherapeutic drugs, and have often undergone an epithelial-to-mesenchymal transition (EMT; see Glossary, Box 1) (Singh and Settleman, 2010). Dicer (Iliou et al., 2014) and multiple miRNAs [including, miR-34a (Bu et al., 2013, 2016), miR-106b (Zheng et al., 2015a), miR-140 (Zhai et al., 2015), miR-146a (Hwang et al., 2014), miR-183 (Wellner et al., 2009), miR-200 (Wellner et al., 2009), miR-203 (Wellner et al., 2009), miR-215 (Jones et al., 2015), miR-302b (Zhu et al., 2012), miR-328 (Xu et al., 2012b), miR-363 (Tsuji et al., 2014), miR-371 (Li et al., 2015c) and miR-451 (Bitarte et al., 2011)] reportedly regulate CRC TICs. As part of a positive-feedback loop (a recurring phenomenon with miRNAs in cancer), the E-box transcription factor and inducer of EMT, SNAIL, promotes β-catenin-mediated transcription of miR-146a (Hwang et al., 2014). β-catenin acts in the nucleus as a transcriptional co-activator with DNA-binding transcription factors from the TCF/LEF family, most frequently as a downstream mediator of Wnt signaling (MacDonald et al., 2009). This activation of miR-146 promotes symmetric division of TICs by targeting and inhibiting NUMB, a protein that negatively regulates Notch (see Glossary, Box 2) and the stability of β-catenin (Hwang et al., 2014). Symmetric stem cell division occurs when one stem cell creates two daughter stem cells, whereas asymmetric division generates one stem cell and one progenitor committed to differentiation, although with cancer, such ‘progenitors’ do not necessarily differentiate, but instead lack tumor-initiating properties (Meacham and Morrison, 2013). Thus, symmetric divisions of TICs produce more cells that are capable of initiating new tumors and that may be more resistant to chemotherapeutic drugs. Interfering with this regulatory loop not only reduces symmetric divisions (and promotes asymmetric divisions) of CRC TICs, but also restores the susceptibility of cancer cells to cetuximab, an inhibitor of EGFR that is used to treat CRC (Hwang et al., 2014).

Conversely, miR-215, which is transcriptionally activated by the intestinal-specific transcription factor CDX1, appears to repress stem cell markers and is significantly depleted in CRC TICs (Jones et al., 2015). MiR-215 (and the related miR-192), functions in part by regulating cell cycle genes and by repressing cell proliferation (Boni et al., 2010; Fesler et al., 2015; Jones et al., 2015). This is consistent with the prognostic features of miR-192 (Chiang et al., 2012) and miR-215 (Karaayvaz et al., 2011; Faltejskova et al., 2012; Chiang et al., 2012; Li et al., 2013b), which are both depleted in CRC tumors.

**Fig. 3.**
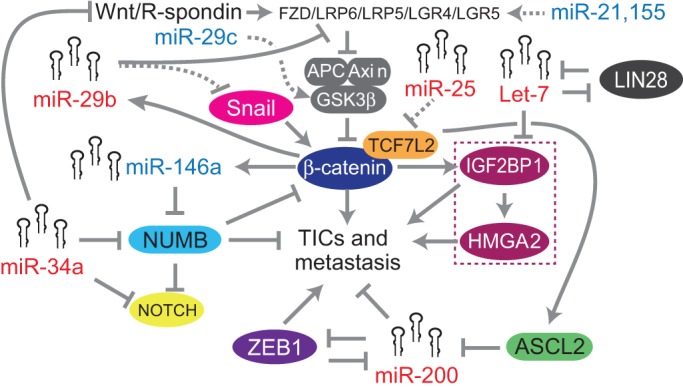
**Relationships among miRNAs and canonical Wnt signaling, metastasis, and tumor-initiating cells.** The canonical Wnt signaling pathway is activated via Wnt and R-spondin interaction with Frizzled (FZD) receptors together with LRP5/LRP6 co-receptors and LGR4/LGR5 co-activators (depicted at the top of the figure). This causes inhibition of the APC-Axin-GSK3β complex, leading to the stabilization of β-catenin, which interacts with TCF7L2 (previously TCF4; shown in orange). This triggers the transcriptional activation of target genes. This pathway also enhances a stem cell phenotype in intestinal epithelial cells and drives metastasis of tumor-initiating cells (TICs). Several miRNAs directly modulate canonical Wnt signaling or other effectors, such as NUMB, NOTCH and ASCL2. Oncogenic miRNAs are depicted in blue, tumor-suppressive miRNAs in red. Direct relationships are shown with solid lines, indirect relationships with dotted lines. Official human gene symbols and full names: *RSPO1-RSPO4*, R-spondin 1-4; *FZD1*-*FZD10*, frizzled class receptor 1-10; *LRP5/6*, LDL receptor related protein 5/6; *LGR4/5*, leucine-rich repeat containing G protein-coupled receptor 4/5; *SNAI1*, Snail family transcriptional repressor 1; *NUMB*, endocytic adaptor protein; *APC*, adenomatous polyposis coli or Wnt signaling pathway regulator; *AXIN1/2*, axin 1/2 ; *GSK3B*, glycogen synthase kinase 3 beta; *TCF7L2*, transcription factor 7 like 2 (previously TCF4); *CTNNB1*, β-catenin; *ZEB1*, zinc finger E-box binding homeobox 1; *LIN28A* and *LIN28B*, lineage-28; *IGF2BP1*, insulin-like growth factor 2 mRNA binding protein 1; *HMGA2*, high mobility group AT-hook 2; *ASCL2*, achaete-scute family bHLH transcription factor 2.

Less is known about the regulation of normal intestinal stem cells by miRNAs. Nonetheless, recent studies have demonstrated that both miR-34a and Let-7 (see Glossary, Box 1) miRNAs appear to repress stem cell fate in the gut (Bu et al., 2016; Madison et al., 2015). Like miR-146, miR-34a modulates stem cell fate through the inhibition of *NUMB* [which inhibits Notch, a positive regulator of IESC fate (Pellegrinet et al., 2011)], although miR-34a inhibits symmetric stem cell division, probably because of its effects on an additional target of miR-34a, *Notch1* (Bu et al., 2016). It is also worth noting that miR-34a represses IESC fate only in response to inflammatory signals (Bu et al., 2016), elaborated further below. Inhibiting miRNAs that promote TIC activity in CRC might prove beneficial for eradicating this cancer cell population or for conferring therapeutic sensitization, although deleterious effects on the normal IESC population must be avoided. Moreover, the possible plasticity of cellular identity may render TICs as an ever-moving target (Meacham and Morrison, 2013), complicating the elimination of a specific TIC lineage. Examples of how miRNAs regulate CRC TICs are shown in Fig. 3.

Regulators of miRNA biogenesis (see Box 2) can also modulate stem cell function and tumorigenesis. This is observed for the RNA-binding proteins LIN28A and LIN28B, which directly repress the biogenesis of Let-7 miRNAs. As discussed in more detail later in this Review, LIN28 proteins appear to play an oncogenic role in many cancer types (Viswanathan et al., 2009), including CRC (King et al., 2011a,b). Several reports, including our own, have documented the pro-tumorigenic effects of LIN28 proteins on the initiation and progression of intestinal tumorigenesis in mouse models (Madison et al., 2013; Tu et al., 2015). LIN28B also augments intestinal organoid colony-forming potential, which is a stem cell phenotype (Madison et al., 2013). This suggests that LIN28B enhances, while Let-7 represses, IESC fate, symmetric division or survival. Spontaneous tumors in transgenic mice that overexpress LIN28 in the intestine also exhibit hyperactivation of the Wnt signaling pathway (Madison et al., 2013; Tu et al., 2015). These oncogenic effects were found to be largely due to the LIN28-mediated depletion of Let-7 miRNAs (Madison et al., 2013; Tu et al., 2015).

Thus, miRNAs can interact with known stem cell signaling pathways, such as Notch and Wnt, to augment stem cell fate or proliferation, which could contribute to development of CRC. The direct targets and relevant mediators downstream of miR-146 and miR-34a implicate the Notch and Wnt signaling pathways, while regulators remain to be delineated downstream of Let-7, excepting recent insights into a Let-7 target, *Hmga2*, which is discussed later in this Review.

## MicroRNA interactions with known molecular drivers of CRC

Known drivers of CRC have been identified through large-scale cancer genome sequencing efforts (Cancer Genome Atlas Network, 2012; Guinney et al., 2015) and include frequent oncogenic mutations in *KRAS*, *BRAF* and *TP53*, which are also common in other cancer types, such as melanoma (Krauthammer et al., 2012), and malignancies of the lung (Cancer Genome Atlas Research Network, 2014) and pancreas (Bailey et al., 2016). However, the hallmark feature of CRC is the hyperactivation of the Wnt pathway, usually caused by mutations in the tumor suppressor gene *APC*. Below, we discuss the interaction of miRNAs with genes commonly mutated in CRC, beginning with the relationships of miRNAs with Wnt pathway regulators.

### Wnt signaling modulation by miRNAs

The deregulation of Wnt signaling is the most frequent molecular aberration in CRC, with inactivating mutations in the *APC* gene occurring in ∼75% of all tumors (Cancer Genome Atlas Network, 2012; Guinney et al., 2015). *APC* encodes a large scaffolding protein that is part of the AXIN destruction complex, which is necessary for the phosphorylation and degradation of β-catenin (MacDonald et al., 2009). Loss of *APC* function results in increased levels of β-catenin, a key effector of Wnt signaling that interacts with the HMG-box DNA-binding factor TCF4 (TCF7L2) to drive target gene transcription. Activating mutations in *CTNNB1* (β-catenin; see Glossary, Box 1), or in other Wnt signaling activators (e.g. *RSPO2/3*) (Morin et al., 1997; Seshagiri et al., 2012) can also hyperactivate Wnt signaling in CRC, as can inactivating mutations in Wnt repressors (e.g. *AXIN2*, *SFRP1*, *RNF43* or *ZNRF3*) (Liu et al., 2000; Suzuki et al., 2004; Koo et al., 2012). Alternatively, miRNAs can also modulate Wnt signaling through the repression of pathway components. For example, miR-135a/b miRNAs, which are overexpressed in CRC, are able to directly target *APC*, leading to the upregulation of Wnt signaling (Nagel et al., 2008). MiR-135a/b is also predicted to target and inhibit secreted frizzled-related protein 4 (SFRP4), which binds and represses extracellular Wnt proteins (Kawano and Kypta, 2003). *SFRP4* and miR-135 expression is inversely correlated in multiple types of cancer (Jacobsen et al., 2013). In a likely positive feedback loop, miR-135b is transcriptionally activated by TCF4/β-catenin, and is dramatically increased in colonic tumors in mice with inactivated *Apc* and in sporadic human CRC (Valeri et al., 2014). In mice, mutations in the canonical CRC tumor suppressors *Pten* or *Trp53* in the context of *Apc* loss greatly enhance the upregulation of miR-135b, with phosphoinositide 3-kinase (PI3K) and downstream FOXO1 and FOXO3A transcription factors confirmed as regulators of miR-135b (Valeri et al., 2014). The mechanism of TP53-mediated repression of miR-135b, however, has yet to be explored. In addition, given that the inhibition of miR-135b by specific antagomiRs greatly represses tumorigenesis in multiple mouse models (Lin et al., 2013; Valeri et al., 2014), miR-135b might drive tumorigenesis by inhibiting multiple tumor suppressors (besides *APC*), such as *TGFBR2* (Valeri et al., 2014). Known relationships between canonical Wnt signaling and miRNAs are illustrated in Fig. 3.

Also operating upstream of Wnt, the miR-34 family (miR-34a/b/c) directly targets and represses multiple effectors of Wnt signaling, including WNT1, WNT3, LRP6 (a Wnt ligand co-receptor), β-catenin and LEF1 (an HMG-box transcription factor that, like TCF4, interacts with β-catenin) (Kim et al., 2011). The transcription of miR-34 is directly stimulated by TP53, providing insight into how TP53 can repress Wnt signaling (Kim et al., 2011). This relationship might be integral to the tumor-suppressive properties of miR-34 miRNAs; the negative regulation of Wnt signaling might also mediate miR-34-driven repression of intestinal stem cell fate (see below). Additional inhibitors of Wnt ligands, or β-catenin-mediated function, include miR-29b, miR-29c, and miR-93, which target BCL9L [a β-catenin co-activator and miR-29b target (Subramanian et al., 2014; Zhang et al., 2014b; Tang et al., 2015)], GNA13 and PTP4A [miR-29c targets that negatively regulate GSK3β (Zhang et al., 2014b), a kinase that phosphorylates β-catenin and triggers degradation] and SMAD7 [a miR-93 target that promotes nuclear accumulation of β-catenin (Subramanian et al., 2014; Zhang et al., 2014b; Tang et al., 2015)]. Although the precise mechanism is not known, miR-21 can also enhance TCF4/β-catenin-mediated transcriptional activation of surrogate reporters (i.e. TOPFLASH) and endogenous target genes. This miR-21 activity is coincident with, and dependent on, β-catenin phosphorylation at Ser552 (Lin et al., 2014). In another example of a potential positive feedback loop, miR-21 might be directly activated by TCF4/β-catenin. This possibility is based on the identification of TCF4 binding sites near miRNA transcriptional start sites, with confirmation of binding via chromatin immunoprecipitation (ChIP) and qPCR in CRC cell lines (Lan et al., 2012). The pro-oncogenic miRNA, miR-155, might also enhance Wnt/β-catenin signaling by directly targeting and inhibiting *HMGB1*, which has to date only been reported as a factor that promotes Wnt signaling (Itou et al., 2011; Zhou et al., 2012; Wan et al., 2016).

In summary, many miRNAs act as regulators of the Wnt pathway at multiple levels of the signaling cascade, and some miRNAs, such as miR-34, are capable of restraining both Wnt and Notch signaling pathways. Wnt-modulating miRNAs deserve particular scrutiny as potential therapeutic targets given that the Wnt pathway is a central oncogenic driver of CRC. However, additional mutations in genes such as *KRAS* and *BRAF* are also required in CRC, indicating that multiple pathways are therapeutically relevant.

### miRNA modulation of the small GTPases KRAS and BRAF

Nearly half of all CRC tumors have activating mutations in either of the small GTPases, *KRAS* and *BRAF*, which activate the MAP kinase (MAPK) pathway and stimulate cell proliferation (Cancer Genome Atlas Network, 2012; Guinney et al., 2015). Although studies of some cancers, such as lung and breast cancer, have demonstrated that Let-7 miRNAs directly repress *KRAS* mRNA (Esquela-Kerscher et al., 2008; Iliopoulos et al., 2009; Johnson et al., 2005), *KRAS* has not been found to be a Let-7 target in studies of CRC (King et al., 2011b; Madison et al., 2013, 2015). In contrast to Let-7, miR-31 appears to be a potent stimulator of KRAS in CRC, via negative regulation of *RASA1*, an inhibitor of KRAS function (Kent et al., 2016; Sun et al., 2013).

The *BRAF* oncogene is frequently mutated in tumors located in the proximal colon and is implicated in driving microsatellite instability (MSI) colon cancers (Domingo et al., 2004). MSI colon cancers are hypermutated (see Glossary, Box 1), probably as a result of methylation or mutation of the *MLH1*, *MSH2* or *MSH6* genes, which encode proteins necessary for DNA mismatch repair (Richman, 2015). Although *BRAF* has not been shown to have a direct relationship with many miRNAs, one report has characterized *BRAF* mRNA as a target of the miR-378 anti-oncomiR in CRC (Wang et al., 2015c). More frequently, high miR-31 expression is associated with *BRAF* mutations and an aggressive cancer phenotype (Ito et al., 2014; Nosho et al., 2014; Choi et al., 2016). However, our present understanding of the molecular function of miR-31 is limited, although this oncomiR is reported to be transcriptionally activated downstream of the KRAS-BRAF-MAPK signaling cascade (Kent et al., 2016).

### The miR-34 anti-oncomiR family and TP53

The miR-34 family consists of miR-34a, transcribed at one locus, and miR-34b and miR-34c, co-transcribed at another locus. These miRNAs regulate mRNAs involved in the cell cycle (Ebner and Selbach, 2014), growth (Kress et al., 2011), DNA damage (Takeda and Venkitaraman, 2015) and apoptosis (Ebner and Selbach, 2014); these interactions are likely to be associated with the tumor-suppressive properties of miR-34 miRNAs and their ability to induce apoptosis and senescence (Tazawa et al., 2007). The miR-34a and the miR-34b/c loci are direct transcriptional targets of the TP53 tumor suppressor (Chang et al., 2007; He et al., 2007; Raver-Shapira et al., 2007; Tarasov et al., 2007), the levels of which, in turn, are indirectly augmented by miR-34. This positive feedback on TP53 is likely to be due to several mechanisms. First, miR-34 directly represses *Mdm4* (*HDM4* in humans), which encodes a RING-finger protein that binds to TP53 and blocks its ability to activate target genes (Okada et al., 2014). Second, miR-34 promotes modest (and probably indirect) stimulation of the *TP53* promoter (Gao et al., 2015). Independent of TP53, miR-34 is induced by FOXO3A (forkhead box O3a transcription factor) in a feedback loop involving MK5 and MYC. *MK5* is a MYC target gene that encodes a kinase that phosphorylates and activates FOXO3A, which then directly activates the expression of the miR-34b/c promoter (Kress et al., 2011). MK5 is downregulated in CRC, and is associated with reduced survival in CRC patients (Kress et al., 2011). Thus, both MYC and TP53 can promote miR-34 expression.

Another key role for miR-34a in CRC is the regulation of IL-6/STAT3 signaling (see Glossary, Box 1), which fuels EMT and metastasis in CRC (Rokavec et al., 2014). In this pathway, STAT3 directly represses the transcription of miR-34a, which in turn, directly represses the IL-6 receptor (*IL6R*), thus forming a positive feedback loop (Rokavec et al., 2014) (Fig. 2). Using cell culture models and inflammatory bowel disease (IBD) mouse models, Rokavec et al. (2014) revealed that miR-34a is key for the repression of tumor migration, invasion and metastasis via repression of the IL-6/STAT3 signaling pathway. Studies using cultured human CRC cells (Bu et al., 2013) and mouse models (Bu et al., 2016) indicate that miR-34 also regulates IESC and CRC stem cell division. Relevant to IESC, the epithelial inactivation of miR-34a does little to perturb stem cell proliferation in the mouse intestine (Bu et al., 2016). However, in the context of inflammatory stimuli [i.e. DSS (dextran sulfate sodium)-induced colitis mouse models or in response to TNFα treatment; see Glossary, Box 1], the loss of miR-34a enhances symmetric IESC division (Bu et al., 2016). Interestingly, TP53 has a similar effect; in mouse models, inactivation of *Trp53* (the mouse homologue) enhances IESC competition and clonal expansion exclusively in the context of inflammation (Vermeulen et al., 2013). Likewise, in humans, an inflammatory microenvironment enables mutations in *TP53* to accelerate clonal expansion of pre-cancerous lesions, promote tumor growth and fuel cancer progression in CRC (Brentnall et al., 1994; Leedham et al., 2009).

Thus, although miR-34a may not be required for TP53 function in the context of normal TP53 dosage (Okada et al., 2014), miR-34a deficiency appears to augment CRC tumorigenesis when TP53 is haploinsufficient. Aside from acting as an effector of TP53, miR-34a expression downstream of the canonical oncogenic transcription factors, MYC and STAT3, described above, provides negative feedback on tumor cell proliferation, survival and metastasis, which highlights the multifaceted mechanisms by which miR-34 represses tumorigenesis. The connection of miR-34 with TNFα and IL-6 also highlights the key role of inflammation, which increases the risk and fuels the progression of CRC (Lasry et al., 2016).

## miR-21 and miR-221/222 in pro-inflammatory signaling pathways

miR-21 is one of the most prominent oncomiRs in CRC, and has demonstrated pro-tumorigenic properties in many other solid tumor types (Pan et al., 2010; Wang et al., 2014c). Repression of the well-characterized target PDCD4, a tumor suppressor, appears to mediate the key oncogenic effects of miR-21 (Frankel et al., 2008). PDCD4 is a pro-inflammatory factor that is activated by apoptosis stimuli and is required for the lipopolysaccharide (LPS)-mediated activation of NF-κB (NFΚB) and IL-6 (see Glossary, Box 1) (Sheedy et al., 2010). PDCD4 can also be repressed by PGE2 (see Glossary, Box 1), a pro-inflammatory prostaglandin that also activates Wnt signaling (Buchanan and DuBois, 2006). COX2, a prostaglandin-endoperoxide synthase, is responsible for generating PGE2 and is frequently overexpressed in CRC (Sano et al., 1995). Studies indicate that COX2/PGE2-mediated repression of PDCD4 occurs via the induction of miR-21 (Peacock et al., 2014). Consistent with this, PDCD4 protein levels decrease progressively during CRC tumorigenesis, as normal tissue transforms to adenocarcinoma (Ma et al., 2013a; Mudduluru et al., 2007) and loss of PDCD4 protein is significantly associated with reduced patient survival (Mudduluru et al., 2007). PDCD4 also represses the invasion and intravasation of CRC cell lines in a chick embryo metastasis model (Asangani et al., 2008); there is evidence that PDCD4 executes this function by repressing the pro-metastatic urokinase receptor (uPAR) (Leupold et al., 2007).

MiR-21 is also activated downstream of NFΚB and MyD88, an adapter of Toll-like receptors (TLRs) needed for NFΚB activation by TLR ligands such as LPS (Kawai et al., 1999). MiR-21 appears to be integral to the inflammation observed in colitis-associated colon cancer: in a carcinogen-induced mouse model of CRC using the mutagen azoxymethane (AOM) plus DSS, genetic inactivation of miR-21 reduced tumor burden and decreased levels of pro-inflammatory cytokines (Shi et al., 2015). Loss of miR-21 in tumors in this model also increased apoptosis and levels of PDCD4, and reduced levels of activated STAT3 and BCL2 (Shi et al., 2015). This is consistent with a role for PDCD4 in promoting apoptosis. In addition to PDCD4, the involvement of other miR-21 targets in tumorigenesis seems likely. In the breast cancer cell line MCF10A, miR-21 activation (via repression of PTEN, a PI3K antagonist) appears to be necessary for the optimal activation of NFΚB, which leads to a positive feedback loop that activates the expression of IL6 and STAT3, which directly activates the transcription of miR-21 (Iliopoulos et al., 2010). Reminiscent of this function of miR-21 in MCF10A, miR-221 and miR-222 augment NFΚB and STAT3 by indirectly modulating their protein stability through miR-221/222-mediated direct inhibition of the nuclear E3 ubiquitin ligase *PDLIM2* (Liu et al., 2014). MiR-221 also targets and inhibits *PTEN* (Xue et al., 2013) and the anti-metastatic factor *RECK* (Qin and Luo, 2014). Consistent with this, overexpression of miR-221 is associated with lymph node metastasis of CRC (Hur et al., 2015).

Overall, inflammatory signaling pathways are key drivers of CRC (Lasry et al., 2016), and miR-21 appears to be a key modulator of several pro-oncogenic and immunomodulatory factors, such as PDCD4, NFΚB, and STAT3. Less is known about the involvement of miR-221/222; however, these miRNAs might also be key enhancers of NFΚB and STAT3 activation. Repressing the activity or expression of these miRNAs, especially miR-21, which is frequently overexpressed in CRC (Slaby et al., 2007; Yamamichi et al., 2009) and is associated with poor outcomes (Chen et al., 2016), could represent an effective therapeutic strategy for CRC.

## The miR-17 oncomiR family and modulation of TGF-β signaling

The human miR-17 family consists of eight miRNAs (miR-17, miR-18a/b, miR-20a/b, miR-93, and miR-106a/b), with three of these (miR-17, miR-18a, and miR-20a) transcribed from the miR-17-92 miRNA locus. MiR-17 appears to promote CRC tumorigenesis, evident in studies showing that it suppresses apoptosis and cell cycle arrest, increases migration, and drives tumor xenograft growth of CRC cell lines (Ma et al., 2012). Further analysis has revealed that miR-17 directly represses the cell cycle regulator and RB-family member P130 (RB transcriptional co-repressor like 2, *RBL2*), a tumor suppressor that also negatively regulates β-catenin levels and Wnt signaling (Ma et al., 2012). MiR-17 also targets and inhibits *PTEN* (Fang et al., 2014) and RHOE (*RND3*), which encodes a GTP-binding protein (without GTPase activity) that is reduced in CRC and can repress tumor cell invasion (Thuault et al., 2016), promote contact inhibition (Hernández-Sánchez et al., 2015) and downregulate Notch signaling (Tang et al., 2014b; Zhu et al., 2014b).

Other miR-17 family members are also modulators that fuel cancer progression. MiR-20a promotes CRC cell line migration, invasion and the expression of EMT markers (Sokolova et al., 2015; Xu et al., 2015; Cheng et al., 2016). There is evidence that miR-20a promotes cell cycle progression in response to the multifunctional cytokine, transforming growth factor beta (TGF-β), a pro-metastasis but growth-repressive cytokine that represses *MYC* expression, induces p21 (*CDKN1A*), and delays entry into G1/S phase. The direct repression of *CDKN1A* and constituents of a MYC-repressing complex (consisting of E2F5 and KLF11) by miR-20a were identified as key interactions contributing to the ability of miR-20a to neutralize the growth-repressive properties of TGF-β (Sokolova et al., 2015). MiR-20a abrogation of the growth repression by TGF-β might enhance the ability of TGF-β to drive migration, invasion and cancer cell metastasis (Oft et al., 1998).

Like miR-20a, miR-106a/b also appear to enhance metastasis or an EMT phenotype. MiR-106a targets the TGF-β recteptor *TGFBR2* and is highly expressed in metastatic CRC cell lines, and promotes cancer cell migration and invasion *in vitro* (Feng et al., 2012). TGFBR2 repression might be important for facilitating cell proliferation early in tumorigenesis, but also later in metastasis to facilitate a mesenchymal-to-epithelial (MET) transition (see Glossary, Box 1). Alternatively, and perhaps unexpectedly, given that TGF-β generally enhances EMT (Oft et al., 1998; Lamouille et al., 2014), TGFBR2 may repress migration and invasion, as observed by Feng et al. (2012), although such a role remains to be functionally dissected. Oddly, miR-106b has been reported to have both stimulatory (Feng et al., 2012; Zhang et al., 2015a) and inhibitory (Zheng et al., 2015a) effects on the migration and EMT of CRC cell lines. In contrast, there are concordant findings that miR-106b promotes CRC tumor cell metastasis (Feng et al., 2012; Zhang et al., 2015a; Zheng et al., 2015a), although one study implicated the anti-metastatic factor *DLC1* (Zhang et al., 2015a) as the relevant miR-106b target, while the other implicated *PRRX1* (Zheng et al., 2015a). The mechanisms underlying the downstream effectors of miR-106b demand further scrutiny, in light of these disparate findings.

Further studies are certainly needed to investigate the roles of the miR-17 family miRNAs in CRC, particularly miR-18a, which is reported to be elevated in CRC (Song et al., 2011; Wu et al., 2013). MiR-18a contributes to DNA damage, but can reduce the proliferation of CRC cell lines and enhance their sensitivity to apoptotic stimuli (for example, in response to etoposide, a topoisomerase inhibitor and chemotherapeutic drug) following its forced expression (Wu et al., 2013; Humphreys et al., 2014). The contribution of miR-18a to DNA damage and apoptosis is reportedly due to its direct inhibition of the ATM kinase, which is required for initiating DNA repair following double-stranded breaks (Song et al., 2011; Wu et al., 2013). In summary, studies to date indicate that the miR-17 family fuels CRC metastasis, with interaction with TGF-β signaling as well as other pathways that modulate EMT.

## The miR-10 anti-oncomiR family

The miR-10 family consists of seven miRNAs (miR-10a/b, miR-99a/b, miR-100 and miR-125a/b). Most studies of the miR-10 family suggest that these miRNAs possess tumor suppressive properties (Stadthagen et al., 2013; Chen et al., 2014c; Chen and Xu, 2015; Li et al., 2015a), although oncogenic function has also been observed (Nishida et al., 2011, 2012; Wang et al., 2016). In support of the former role, female *Apc^min^* mice develop significantly more intestinal polyps on a miR-10a knockout background than do *Apc^min^* mice on a WT background (Stadthagen et al., 2013). Interestingly, male *Apc^min^* mice do not display this effect. This sexual dimorphism is possibly due to the observed increase of lactoperoxidase (LPO), an enzyme that can metabolize estrogens into depurinating mutagens. *Lpo* is transcriptionally activated by the transcription factor KLF5, a target of miR-10a that is also depleted in *miR10a^–^*^/–^/*Apc^min^* intestine. However, LPO levels are only elevated in the colon of *miR10a^–^*^/–^/*Apc^min^* mice, yet tumor multiplicity is most evident in the small intestine, suggesting that other pathways contribute to tumorigenesis downstream of miR-10a (Stadthagen et al., 2013).

*In vitro* studies of CRC cell lines indicate that miR-100 and miR-125b promote apoptosis, and thus may repress tumorigenesis, or may be important for the sensitization of tumors to chemotherapeutic drugs (Gong et al., 2013; Peng et al., 2014). In contrast, miR-10b is upregulated in CRC, is associated with metastasis and can repress the pro-apoptotic protein *BIM* (Nishida et al., 2012). In CRC, miR-10b upregulation might promote migration and invasion through the direct repression of *HOXD10* (Wang et al., 2016). The repression of HOXD10 in turn stimulates the increased expression of RHOC, a pro-metastatic small GTPase that has been implicated in metastatic breast cancer (Ma et al., 2007). However, despite expectations that miR-10b would be elevated, levels of miR-10b are only subtly increased in CRC tumors compared with normal intestinal tissue (Wang et al., 2016), consistent with miRNA-sequencing studies by The Cancer Genome Atlas (TCGA) project by the National Cancer Institute (Jacobsen et al., 2013). Moreover, miR-10b levels are not globally increased in metastatic breast cancer (Gee et al., 2008). If elevated miR-10b levels drive tumorigenesis, this might occur only in a distinct subpopulation of tumor cells, as hypothesized for breast cancer (Gee et al., 2008) and as observed in circulating CRC cells (Gasch et al., 2015), or miR-10b might play a temporally limited role at a particular stage of tumorigenesis. Indeed, one study has reported that miR-10b is depleted in CRC-associated liver metastases, while its upregulation in primary CRC tumors is predictive of distant metastasis (Hur et al., 2015). This suggests that miR-10b might play a stage-specific role in the metastasis of tumor cells, perhaps via EMT, while also having a counter-productive effect in the establishment of metastases. This situation is reminiscent of the effects that miR-200 has on breast cancer metastasis, whereby miR-200 promotes MET to facilitate metastasis (Korpal et al., 2011).

The miR-10 family certainly deserves further examination to determine how individual members affect CRC tumorigenesis, especially in light of the possibly pleiotropic effects or stage-specific functions described above. Moreover, a role for miR-10 miRNAs in regulating metastasis seems evident, but needs further scrutiny. One odd but significant feature of the arrangement of the miR-10 gene family is that the miR-99, miR-100, and miR-125 genes (all except miR-10a/b) are physically clustered with the loci that encode Let-7 miRNAs. One can certainly speculate that chromosomal deletions or the transcriptional silencing of these miR-10 genes might also affect Let-7 miRNAs as well, although this has not yet been documented.

## Let-7 and CRC tumorigenesis

Let-7 miRNAs account for 4 out of the 15 most abundant miRNAs in mouse intestine (McKenna et al., 2010). Many functional studies of Let-7 in CRC have uncovered important roles for these miRNAs through overexpression of the specific and potent Let-7 inhibitors LIN28A and LIN28B. In CRC cell lines, LIN28B overexpression enhances migration and invasion, and these responses depend on the depletion of Let-7 miRNAs by LIN28B (King et al., 2011b). Studies in LIN28 transgenic mice have revealed that Let-7 miRNAs are critical for repressing tumorigenesis in the intestine (Madison et al., 2013, 2015; Tu et al., 2015). *In vivo* genetic studies of Let-7 function are lacking, probably because of high expression levels in the intestine (McKenna et al., 2010) and redundancy (Abbott et al., 2005) of this large miRNA gene family. Despite these hurdles, we recently depleted all Let-7 miRNAs in the mouse intestine epithelium by tissue-specific expression of LIN28B in conjunction with an intestine-specific knockout of the *Mirlet7c2*/*Mirlet7b* cluster (Madison et al., 2013, 2015). These genetic manipulations caused the spontaneous development of adenocarcinomas with a high penetrance, a phenotype that depends on the Let-7 target *Hmga2*, a DNA-binding non-histone high mobility group chromatin protein and likely oncogene (Madison et al., 2015). Aside from *Hmga2* (Boyerinas et al., 2008; Gurtan et al., 2013), Let-7 miRNAs also repress *IGF2BP1*,* IGF2BP2*, *TRIM71* (which encode RNA-binding proteins) and *NR6A1* (an orphan nuclear receptor) (Boyerinas et al., 2008; Gurtan et al., 2013). Among these, *IGF2BP1* is a transcriptional target of Wnt-TCF4/β-catenin signaling and promotes tumorigenesis and metastasis in CRC cell lines and mouse models (Noubissi et al., 2006; Hamilton et al., 2013).

Let-7 miRNAs are among the most important miRNAs for repressing tumorigenesis because of their abundance, anti-proliferative function and pro-differentiation effects. Therefore, augmenting Let-7 miRNA function may be key for the prevention or treatment of CRC. Despite the abundance and redundancy of the 12-member Let-7 miRNA gene family, they are largely susceptible to the overexpression of either LIN28A or LIN28B in CRC, which can function as potent oncogenes (Viswanathan et al., 2009). In other cancers LIN28B can be induced by oncogenic drivers that are activated in CRC – by NFΚB (Iliopoulos et al., 2009) and Wnt signaling (Cai et al., 2013) in breast cancer and by MYC in a B-cell lymphoma cell line (Chang et al., 2009) – although direct relevance to CRC remains to be determined.

## miR-15 anti-oncomiR family

The miR-15 family (miR-15, miR-16, miR-195) can also include miR-424 and miR-497. MiR-16 and miR-195 are depleted in CRC tumors relative to normal tissue (Wang et al., 2012; Qian et al., 2013; Xiao et al., 2014). Mechanistically, the miR-15a/miR-16-1 locus is induced by TP53 in response to DNA damage and is responsible for directly repressing the pro-metastatic bHLH transcription factor AP-4 (*TFAP4*) in CRC cells (Shi et al., 2014). Shi et al. (2014) demonstrated using mouse xenograft models of lung metastasis that the repressive effects of miR-15a/16 on migration, invasion, and EMT are due to their direct repression of *TFAP4*. As is frequently observed for miRNAs, the authors also uncovered a negative feedback loop, whereby TFAP4 directly represses the transcription of the miR-15a/miR-16-1 locus.

## Pleiotropic effects of miR-200 miRNAs

Multiple miRNAs appear to have pleiotropic or context-dependent effects in CRC. Such effects are not wholly understood, except in the case of the miR-200 family. This family is often reported as an important factor in maintaining epithelial identity and in repressing EMT (Zaravinos, 2015); however, miR-200 miRNAs can also promote metastasis, through effects on MET – a process that can promote the establishment of a metastasis (Korpal et al., 2011; Diepenbruck and Christofori, 2016). The miR-200 family consists of miR-200a/b/c, miR-141 and miR-429, which are located in two gene clusters in mice and humans. These miRNAs repress the pro-mesenchymal transcription factors *ZEB1*, *ZEB2* and *PRRX1* (Park et al., 2008). Consistent with their role in repressing growth and EMT in primary tumors, miR-200 miRNAs are downregulated in CRC tumors via promoter methylation, and they function in a cell-autonomous manner in tumor epithelium (Davalos et al., 2012). Definitive studies demonstrating a pleiotropic role for miR-200 in CRC are lacking; however, descriptive studies suggest that miR-200 miRNAs might promote the establishment of metastases. Specifically, miR-200c and miR-141 have been shown to be elevated in liver metastases relative to levels in primary CRC tumors (Hur et al., 2013). Serum levels of miR-200c are also elevated in individuals with CRC metastases, although the source of these miRNAs might originate from non-tumor cells (Toiyama et al., 2014). The observed differential methylation of miR-200 promoters between primary tumors and metastases might also reflect the need for plasticity of miR-200 expression (Davalos et al., 2012), depending on the context and tumor environment (Hur et al., 2013). MiR-200 is also regulated by the transcription factor ASCL2, which transactivates the miR-200b-a-429 promoter (Tian et al., 2014). *ASCL2* is a Wnt target gene that promotes stem cell fate in the intestine (van der Flier et al., 2009; Schuijers et al., 2015) and is upregulated in CRC (Jubb et al., 2006). Dynamic levels of *ASCL2*, whose promoter can be methylated and repressed (de Sousa et al., 2011), might also contribute to the context-dependent expression of miR-200.

Despite the evidence that miR-200 miRNAs could promote the establishment of metastasis, such a role remains to be fully examined in CRC. Further studies may require the controlled transgenic expression (or inactivation) of miR-200 miRNAs in animal models to dissect stage-specific or context-specific roles for miR-200.

## miRNAs as diagnostic and prognostic tools

Screening and early detection of cancer is the main approach for CRC prevention in the USA and Europe, in which both fecal occult blood tests (FOBT, see Glossary, Box 1) and colonoscopy are used together or alone, for those with an average risk for CRC at age 50 (Zavoral et al., 2009; Altobelli et al., 2014; Sovich et al., 2015). For individuals with an increased risk, such as those with affected family members, IBD, familial adenomatous polyposis (FAP) or Lynch syndrome, screening by colonoscopy is usually recommended earlier and more frequently. FOBT is a yearly, non-invasive screening technique that detects blood in stool and provides an estimated 24-39% reduction in mortality due to CRC; however, this method suffers from low sensitivity. Often, a positive FOBT results in colonoscopy screening, which is normally recommended every 10 years (Sovich et al., 2015). Colonoscopy is the most prevalent screening method for CRC used in the USA and allows evaluation of both the right and left colon, in addition to the removal of large polyps during the procedure. Although this method reduces the odds of CRC by 30-75%, it is estimated that nearly 25% of polyps are missed during colonoscopy, and it is a costly and invasive technique (van Rijn et al., 2006; Leufkens et al., 2012; Sovich et al., 2015). Given these caveats, cheaper, less-invasive and more quantitative tests would provide an attractive alternative to the current standard screening methods. MiRNAs in blood or stool may provide this alternative.

On a practical level, miRNAs can be used to screen patients for cancer because they are stable and detectable in blood and stool, and their expression profiles reflect their expression in tumors from CRC patients (Dong et al., 2011; Ren et al., 2015). Ideally, screening tests should detect the presence of miRNAs found exclusively in individuals with intestinal adenomas or CRC. Although the use of miRNAs to screen for CRC might never out-perform the preventative successes of routine colonoscopy, their use could offer less-invasive and more cost-effective alternatives to supplement existing screening approaches.

The use of miRNAs for prognostic purposes also holds promise, especially with the implementation of precision medicine in CRC therapy (an approach that takes into account individual variations in gene expression and tumor phenotypes to achieve optimal patient outcomes through tailored therapies). New improvements to miRNA detection technologies such as digital PCR (see Glossary, Box 1) could enable more sensitive methods for the absolute quantification of miRNAs, as recently demonstrated for lung cancer (Li et al., 2014a; Wang et al., 2015a; Campomenosi et al., 2016). The expression signatures of multiple miRNAs and mRNAs could also provide effective diagnostic and prognostic assays for CRC (Li et al., 2014a; Conte et al., 2015).

MiR-21 is perhaps the most studied oncomiR implicated in CRC, as previously discussed. Mir-21 is elevated in CRC tumors, with several studies reporting a step-wise increase in its expression as tumors progress to later stages (see Fig. 1) (Kjaer-Frifeldt et al., 2012; Schee et al., 2012; Toiyama et al., 2013). MiR-21 is also increased in the serum and stool of CRC patients and can accurately predict the local tumor invasion depth (T), lymph node involvement (N) and the presence of distant metastases (M) – collectively, the TNM stage (Kulda et al., 2010; Link et al., 2010; Toiyama et al., 2013; Hofsli et al., 2013; Ogata-Kawata et al., 2014; Bastaminejad et al., 2016). In addition, high levels of miR-21 in primary CRC tissues and matched serum samples are associated with large tumor size and with distant metastasis (Toiyama et al., 2014). High miR-21 expression in tumors is also associated with poor response to chemotherapy and decreased disease-free survival (Schetter et al., 2008; Kulda et al., 2010; Shibuya et al., 2010; Oue et al., 2014). Levels of circulating miR-21 nevertheless decrease following CRC tumor resection (Toiyama et al., 2013). These studies demonstrate that miR-21 in serum and stool reflect its levels in CRC tumors; it thus might serve as both a diagnostic and prognostic biomarker by predicting TNM stage, potential metastasis and responsiveness to chemotherapy. However, increased miR-21 serum levels have also been reported for pancreatic, lung and breast cancers (Volinia et al., 2006; Yan et al., 2008; Toiyama et al., 2013), suggesting that stool analysis should be incorporated to enhance the specificity of CRC screening.

The miR-17-92 cluster is involved in driving tumorigenesis, as discussed above. Several members of this cluster, such as miR-17 (Ng et al., 2009; Yu et al., 2012), miR-20a (Schetter et al., 2008; Motoyama et al., 2009; Earle et al., 2010; Yu et al., 2012), miR-92a (Motoyama et al., 2009; Ng et al., 2009; Earle et al., 2010; Tsuchida et al., 2011; Schee et al., 2012; Wu et al., 2012; Yu et al., 2012) and miR-18a (Motoyama et al., 2009; Brunet Vega et al., 2013; Zhang et al., 2013b), are all reportedly increased in CRC tumors and in serum/plasma, with their elevated levels correlating with recurrence and poor prognosis. Importantly, serum levels of miR-18a (Zhang et al., 2013b) and miR-92a (Huang et al., 2010; Wu et al., 2012) miRNAs decrease following tumor resection. Tests of isolated colonic epithelial cells from the stool of CRC patients have also demonstrated the increased expression of the miR-17-92 cluster (Koga et al., 2010). Overexpression of miR-17-92 has also been reported in other malignancies, including hepatocellular carcinoma, leukemia, pancreatic, breast, ovarian and lung cancer (Ohyashiki et al., 2010; Shigoka et al., 2010; Volinia et al., 2006; Mogilyansky and Rigoutsos, 2013). Such a broad expression profile might limit the effectiveness of this miRNA as a specific biomarker for CRC; however, screening for miR-17-92 in the serum and stool of CRC patients could prove to be a useful prognostic indicator.

The oncomiR miR-29a is also upregulated in tumors (Slattery et al., 2011) and in the blood of CRC patients, which can be used to accurately distinguish patients with early CRC and advanced adenomas (Brunet Vega et al., 2013; Dong et al., 2011; Huang et al., 2010; Luo et al., 2013; Wang and Gu, 2012). In CRC fecal samples, miR-29a was specifically elevated in individuals with rectal tumors, but not colonic tumors (Zhu et al., 2016). MiR-29a has also been proposed as a marker for the early detection of colorectal liver metastasis (CRLM), as levels were significantly higher in both tumors and serum of CRLM patients (Wang and Gu, 2012). This miRNA is also elevated in the serum of patients with multiple myeloma (Sevcikova et al., 2013), but has most extensively been studied as a potential diagnostic biomarker for CRC. The use of miR-29a as a prognostic biomarker for CRC is less clear, but an increase in its levels could indicate early metastasis (Wang and Gu, 2012).

MiR-200c is an oncomiR that promotes epithelial fate, but it can also enhance metastasis (Toiyama et al., 2014). The silencing of miR-200c results in apoptosis and decreased invasion in CRC cell lines (Hur et al., 2013; Chen et al., 2014b; Tanaka et al., 2015). This miRNA is upregulated in CRC tumors and is associated with decreased patient survival, but it is not associated with TNM stage (Xi et al., 2006; Zhang et al., 2013b; Chen et al., 2014b). MiR-200c levels are also elevated in the plasma of CRC patients and decrease after the surgical resection of tumors (Zhang et al., 2013b). In another study, serum levels of miR-200c were specifically increased for stage IV CRC compared with other stages and normal controls, and were also increased in lymph node, liver and other distant metastases (Toiyama et al., 2014). Levels of miR-200c in liver metastases correlate with levels in primary CRC tumors, and miR-200c levels are significantly increased in both the metastasis and the primary tumor, relative to normal adjacent colonic tissue (Hur et al., 2013; Toiyama et al., 2014). Together, these studies demonstrate the potential of miR-200c as a marker for distant metastasis in CRC. Increased miR-200c serum levels in CRC patients also reportedly indicate decreased patient survival (Toiyama et al., 2014).

Many studies demonstrate that the evaluation of miRNAs as biomarkers for CRC in blood and stool is promising, although several challenges remain to be addressed. Notably, several of the miRNAs discussed are altered in cancers other than CRC, and this hinders their use as specific biomarkers of CRC (although the ability to detect multiple cancers could also be seen as an advantage). The implementation of techniques such as digital PCR in diagnostic assays might yield greater sensitivity. Digital PCR is able to detect smaller fold changes in miRNA expression than quantitative PCR (qPCR) and is therefore a highly sensitive and precise technique that can be used with clinical samples (Li et al., 2014a; Wang et al., 2015a).

The altered expression of miRNAs has been reported in CRC tumors and might in the future be used as diagnostic tools and prognostic indicators for CRC. The measurement of miRNAs in the blood or stool could complement current screening methods for CRC and might also provide new insights into mechanisms of tumorigenesis and metastasis. In addition, FOBT samples already collected for CRC screening could be utilized for miRNA analysis (Link et al., 2010). Moreover, miRNAs in stool would serve as an ideal test for the early diagnosis of CRC if altered miRNA levels can be detected earlier in this material than in the blood (Muhammad et al., 2014). An assay that examines the expression of both oncogene and tumor suppressor miRNAs might provide the most comprehensive assessment for diagnostic and prognostic purposes. Although the miRNAs discussed in this section are oncogenic, both miR-215 (Chiang et al., 2012; Karaayvaz et al., 2011; Faltejskova et al., 2012; Slattery et al., 2015) and miR-375 (Dai et al., 2012; Faltejskova et al., 2012; Wang et al., 2014d; Xu et al., 2014, 2016) are tumor-suppressive miRNAs that could be used for CRC screening, although an analysis of miRNA levels in blood or stool is needed.

## Conclusions and future directions

As highlighted in this Review, miRNAs are undoubtedly drivers and modulators of CRC tumorigenesis that have considerable potential as biomarkers and therapeutic targets. Consistently, miRNAs are observed to function in positive- or negative-feedback loops (a biological phenomenon that probably extends miRNA function beyond ‘fine tuning’), highlighting their relevance in self-sustaining epigenetic switches that can change or reinforce cellular phenotypes. Despite the numerous studies of miRNAs and extensive analyses of their expression, the role and function of many individual miRNAs in CRC remains poorly understood. The integrated analysis of multiple miRNA targets for a given miRNA is also needed; i.e. the de-regulation of multiple targets by an aberrant miRNA raises the possibility of interaction, cooperation and possibly synergy between co-regulated targets. The role of miRNAs in tumor stroma also deserves more study, especially considering that some miRNAs, such as miR-143 and miR-145, are exclusively expressed in the stroma, with little likelihood of a cell-autonomous role in CRC cells (Chivukula et al., 2014). High levels of miR-21 are also reported in CRC stroma (Vicinus et al., 2013). Together, the use of miRNAs as biomarkers for CRC might provide a new, less-invasive technique to screen for CRC and to help determine prognosis. We propose that a screening panel consisting of multiple miRNAs might provide the most precise and effective screening tool for CRC.
